# The Deformed and Their Mental Characteristics

**Published:** 1861-04

**Authors:** 


### Art. VII.—THE DEFORMED AND THEIR MENTAL CHARACTERISTICS.

Fair forms and mental excellence, do they go together? Are we what our bodies make us? Does the mind answer to the shape of our heads, spines, and limbs? Are the profiles of Cicero or Marcus Aurelius, such as they are represented in the sculptures of the Campidoglio at Rome, emblematic of the talents and virtues so eloquently expressed in the histories of their lives and writings? Or, is the wonderful repose carved on the features of the First Napoleon, the sublime ideal of Austerlitz or St. Helena, Waterloo or Marengo? The chief charm in the countenance of Byron is the poetic fire that beams from his eye and forehead, for the rest of his face is not formed upon the best of models. Byron imagined that there was a strong resemblance between himself and Marcus Aurelius; and, perhaps, at first sight the resemblance is striking; but the nose, mouth, and chin of Aurelius are indicative of the highest moral perfection, whereas those of Byron betray the grossest sensuality.

Thersites is described by Homer as the ugliest man that came to Troy, and Ulysses says he had never met with a more disagreeable creature. He was squint-eyed, or, as Buttman translates it, bandy, with one leg shorter than the other. His head was peaked and partially bald, or scattered over with thin hair. He had a squeaking voice, a spiteful temper, and a saucy mode of speech. His spine was gibbous between the shoulders. The noblest in the camp were the butt of his cynical impertinence, and he was withal a coward. Ulysses struck him with his staff, and Agamemnon upbraided him in public, without effect.*

This accurate description is the earliest we have of diseased spine. It is earlier than that of Hippocrates by five centuries at least. The physiological as well as the psychological characters of the *Iliad* are touched with the hand of a master. The account that Helen gives to Priam, in the third book, is unrivalled as a piece of graphic writing. The scene passes before you, and each person, as he is mentioned, lives, moves, and speaks with the air and manners proper to himself. The fierce Ajax has broad shoulders and a strongly-built frame. Ulysses is short with an expansive chest and a grave deportment; Menelaus is fair-haired and mild in temper; Agamemnon, tall, athletic, and graceful; Achilles, long-legged; and Hector distinguished by his handsome countenance, sparkling eyes, and exact muscular proportions.† The prettiest man among them is Nireus, who, singularly enough, is, like the ugly Thersites, a great coward.‡

The dwarf, if not a hump-back, is a ricket with the chief characteristics of spinal disease. People of diminutive, as well as of gigantic proportions, are seldom more sound in mind than they are in body. Their temper is malicious or stupid, cruel or weak; and their passions are ungovernable and brutal, or they have no passion at all. The salacity of the dwarf is only too well known. Ariosto makes use of this propensity to point one of his stories with the epigrammatic humour so peculiarly his own. The tale turns upon a fair lady, the wife of a handsome Italian, choosing as her paramour a graceless humpback, who treats her as his mistress with disdain, and serves her base passion with the coolest effrontery. If like goes to like, the lady must have been as deformed in taste as the dwarf in person, with whom she took her pastime.§

To which of the two shall we award the meed of merit in power of speech and fancy—to blind Melesegenes, thence Homer called, or to the incomparable Ariosto? Which fatigues us the soonest, the ancient or the mediæval bard? Perhaps, no one can decide but those who have seen the south of France, the land of Orlando Furioso, or the broad Hellespont and the shores of Troy. In

---

* *Il.* ii. 211. Butt. *Lex.* p. 541. † *Il.* iii. 216.

‡ *Il.* ii. 671. § *Orlando Furioso,* Canto xxviii.

point of good taste and fineness of execution, the Greek excels the Italian poet; but the extravaganzas of Ariosto are too good to part with, and the wild fire of his genius never blazes in vain.

The idea of eccentricity of character being allied to eccentricity of form has not escaped the shrewd mind of Sir Walter Scott. In the *Lay of the Last Minstrel,* the elfin page is introduced with a vivacity and precision which leads us to believe that Sir Walter had some living being of the same description in his eye:—

> "Little he ate, and less he spoke,
> Nor mingled with the menial folk;
> And oft apart his arms he tost,
> And often muttered, Lost, lost, lost!
>   He was waspish, arch, and litherlie,
>   But well Lord Cranstoun served he;
> And of his service was full fain,
> For once he had been ta'en and slain,
>   And it had not been for his ministry.
> All between home and hermitage
> Talk'd of Lord Cranstoun's goblin page."
>
> *Canto* ii., 32.

In private practice, it is not unusual to meet with patients like Scott's elfin page, or Ariosto's dwarf. Sometimes it runs in families, particularly in those where marriages have been contracted between kith and kin. Account for it as we may, such connexions are productive of monstrosities, simpletons, or dwarfs. One of the children, a son or daughter, absorbs all the intelligence and strength of the rest. Of the remainder, one is too tall, another too short, a third bow-legged, and a fourth nothing more than a stunted nonentity. Spinal disease, consumption, or insanity is their common property. The medical attendant is seldom absent from their door. As they grow up, the boys become profligates or incapables, who are eventually laid aside by the world and left to shift for themselves. They end by becoming wearisome dependents on their betters, or sink into sots supported upon a pittance doled out to them weekly by some unseen hand. As to the girls, if they marry, they quickly fall into interminable ill-health, and help to fill up that dreary catalogue of ovarian and uterine maladies, of which they hope to be cured at last so long as their husbands have a fee to spare. Their minds suffer with their bodies. Their nervous fancies are real. They are never free from pain. Their home is their hospital, and domestic comfort is at an end. When their means are large, a long life is spent in the pursuit of health and in the gratification of an egotism which amounts to mental aberration.

Many of these cases are met with in children who have sprung from a late marriage or a drunken father. The wine or spirit drinker engenders an ill-health which is singularly visible in his offspring. The puny child, or dwarfish adult, comes of this source. The pale and beardless face that meets us in the busy streets is the unmistakeable evidence of his parentage. Even dogs may be dwarfed by dosing them with alcohol. The functions are arrested and development is stopped. The bony structure suffers the most, although, very likely, the brain and spinal cord take the lead in the course of defective organization.

Misery, mental and bodily, is entailed on the first, second, or third generations, when the breed ceases, if it have not already become extinct in the first. Convulsions and palsy carry off not a few. The rickety live the longest, albeit, they fill up their place in the world with pain and sorrow, a vexation to themselves and a care to all around them. Hence it comes to pass that deformed persons are proverbially disagreeable and perverse, for they cannot keep pace with their companions, while it is impossible for them to live apart, and destitution to lag behind.

In the character of Richard III. all these qualities are well pourtrayed, as he descants upon his own deformity. It evidently had the worst effect upon the whole of his life. He was not formed to amble in a lady's chamber; the dogs barked at him in the streets; and the sight of his own shadow in the sun irritates him to the last degree of virulence. He feels that the world scouts him as an ill-begotten thing, and he vows revenge upon the world in return. He had the opportunity and the power of doing so, and he wreaks his vengeance even to his own cost. The cruel sarcasms he vents against himself, and the stinging consciousness he betrays of his imbecility as a man, remind us of the petulance with which Lord Byron resented the slightest allusion to his club-foot, or shrunk with morbid sensitiveness from the glance of a stranger casually looking towards the spot upon which he was standing. The bodily uneasiness finds a poor relief in uttering sharp sayings and bitter invectives, which create enemies at every word, or make the careless laugh and good men sigh.

It was out of this class that the royal jesters and buffoons used to be selected. They were looked upon with a degree of wonder amounting almost to superstition; and, were it not for the barbarity and ignorance of the age in which they were fostered about the courts of princes and nobles, we might be tempted to regard the custom of retaining them as a dull satire on the favourites of kings.

Hippocrates has already described these pitiable cripples ages ago. Their long backs, short legs, and long arms; their small hands, narrow chests, protuberant larynx, shrill voices, and poking heads, were signs that did not escape his notice. He says, if they are fleshy and plump, they live to be old; but if they are lean, they die early, generally at or before sixty.

The reader will remember the Black Dwarf in the Waverley Novels. Pliny in his *Natural History* tells us* that the celebrated historian, Tacitus, had a brother who was a perfect monstrosity. In three years he grew six feet and nine inches—*in tria cubita triennio adolevisse.* He was able to walk, but in a slow heavy pace, and was dull of apprehension almost to stupidity. He died of sudden spasms, and violent contractions of the nervous system. No likeness of Tacitus himself has come down to us. But if he was like his model Emperor, Vespasian, he had, according to numismatic authority, a vast head, a long back, short legs, and small arms—unmistakeable signs of rachitis, whether they be found in the person of a victorious Roman Emperor, or in that of his not less highly talented admirer, the author of the history of his times, and the acute annalist of his age and manners. Vespasian's head was most remarkable for its prodigious size, and argued a character greatly above or below mediocrity. His talents were entirely of a military kind. He was certainly superstitious, for he cured a deaf man and a paralytic by his imperial touch.† But his sense of the fine arts was dull, since he forgot himself, and fell asleep in the presence of Nero, as that despot was reciting his own verses to the sound of his lute. For this dire offence, Vespasian ran the risk of forfeiting his life, except, adds Tacitus, that his superior genius or destiny reserved him for the conquest of Judæa.‡

Large trunks with short legs are mostly significant of gross dispositions, and, if the head be large, of a relentless and determined character. In *Gil Blas*, the Prime Minister of the King of Spain is described as a deformity of this sort. The portrait comprises too many particulars for it to be otherwise than original. He was a tall man, much above the common size, and he would have been thought fat, even among the corpulent. He was so high-shouldered that he looked like a humpback, although this was not the case; and his head was so large, that it was thrown forwards, and rested on his chest. His hair was black and straight, his visage long, his complexion sallow, his lips compressed, and his chin pointed and projecting. "This was certainly not the figure of a refined gentleman," says Gil Blas, "but he was agreeable enough whenever he pleased, and just the reverse whenever it served his interests, or suited his fancy, to be so. A libertine, an autocrat, and an intriguer, he at last came to ruin."§

---

* Pliny, *Nat. Hist.*, Lib. vii. 12.

† *Tacit. Hist.*, iv. 81. Statim conversa ad usum manus, ac cæco reluxit dies.

‡ *Tacit. Annal.*, xvi. 5.

§ *Gil Blas*, Livre xi. c. 4.

There is a medium size, above or below which *safe* talents are rarely found; and there is also a *safe* complexion, blended of the ruddy, black, and brown. The most energetic persons are of the brown temperament, and those of great action and discernment usually have aquiline noses. Julius Cæsar's was a small slender figure, with a long neck and a round but not a very large head. Nelson and Napoleon were both small men, and the great Duke of Wellington was not large. St. Athanasius was so small, that a young lady shut him up in her wardrobe, and saved him from the emissaries of Constantius, who were in hot pursuit after him throughout Alexandria.* Levi, the Publican, known as St. Matthew, was a very little man; which accounts for his climbing up the sycamore-tree to see what was passing. St. Thomas, the Apostle, has given his name to streets in some of the capitals of Europe, on account of his diminutive stature, as that of *Little St. Thomas Apostle* in the City of London. St. Augustine was also small, and so was his mother Monica, if we may trust the traditional effigies of them both, which we have seen in the crypt of the magnificent cathedral at Bourges, Central France. Æsop was small and humpbacked; and so was that crooked little thing that asked questions, Pope the poet. And Alexander the Great had a wry neck. The great Apostle St. Paul was, if we may trust the Byzantine historian Nicephorus,† crooked and slightly stooping, small in stature, and of a contracted figure, of a fair complexion, bald, and prematurely old.

The incentive in Byron, says Moore, was that mark of deformity on his person, by an acute sense of which he was stung into the ambition of being great.‡

"Deformity is daring.
It is its essence to o'ertake mankind
By heart and soul, and make itself equal—
Ay, the superior of the rest. There is
A spur in its halt movements, to become
All that others cannot, in such things
As still are free to both, to compensate
For stepdame Nature's avarice at first."
*Deformed Transformed.*

Adopting the sentence of the mighty Byron, we may conclude with the words of Lord Bacon, which the poet apparently had in his mind when he penned the foregoing lines:—"That whosoever has anything fixed in his person that doth induce contempt, has also a perpetual spur in himself to rescue and deliver himself from scorn; therefore all deformed persons are extremely bold."§

One of the most able, if not the most highly favoured, of

* *Gibbon*, xxi. † *Nicephorus*, Lib. ii. c. 37.
‡ Moore's *Life of Byron*, p. 306. Murray, 1860. § *Bacon's Essay*, iv.

Louis XIV.'s marshals, was a sickly humpback, the Duke of Luxembourg.

"He had," says Lord Macaulay,* "a huge pointed hump on his back, and was not only very ugly, but very diminutive also. His constitution was of the feeblest kind, and he was at once a valetudinarian and a voluptuary. His morals were none of the purest. He had great qualities, a rare judgment, and a singular presence of mind. Indeed, his sickly and distorted body seemed to derive health and vigour from disaster and dismay. At the battle of Steinkirke, where he was manifestly taken by surprise, the victory was entirely owing to the coolness and intrepidity with which he faced the critical conjuncture, and restored the order of battle."

Many more instances might be quoted—but enough; so severe a disease cannot but inflict a lasting impression on the sufferer. It may be a good, but it is more often an evil impression, that spreads its influence far and wide. The census of 1851 enumerates 409,207 cases of deformity for England and Wales, and of these 90,277 resided in London. The returns from the manufacturing districts speak of distorted spines as all but universal. Nor is the complaint limited to those who are deprived of the comforts of life, for it is just as frequent among the more affluent classes. Infirmity of mind and inaptitude to the common offices of life, and undeveloped puberty in both sexes, are constantly reported. Few, if any of them are fit for the army. Out of 613 recruits, only 238 were approved for service; the rest were rejected as not strong enough to serve in the defence of their country.†

It is the same in France as in England. At Orleans the number of deformities met with is marvellous. Whole families of bandy-legged and humpbacked may be seen walking along the streets of that sunny town. The cathedral on Sundays is thronged with them; they intermarry, and thus propagate the disease. While sitting in the boulevards at Périgueux, the chief town of Perigord, in 1858, three humpbacks passed us in as many minutes. Dwarfs, humpbacks, and squint-eyed abound in the Pyrenees. The Spanish peasantry that cross the border are small and contemptible. The finest figures are those of the Basques women, who may be seen at Bayonne, Bagnères de Bigorre, and various parts of the Basses Pyrénées, carrying pitchers of water on their heads, and tripping along as upright as a dart. Our very kind hostess at the *Hotel du Parc* at pretty little Dijon, was herself a humpback.

The evil, however, is not a modern one. Hippocrates could

---

* Macaulay, vol. iv. p. 277.

† *A Letter to the Working Classes, &c.* By H. Drummond, M.P. London, 1859. Bosworth and Harrison.

never have described it so accurately had it not been common in his days. The cause of it is a deep question, which would require a treatise by itself, although it is not difficult to divine it. Our object, however, in this article has been to show its mental peculiarities and psychological bearing, and to bring before the profession and the public the consideration of a question which concerns the domestic, the political, and the sanitary condition of the population in the highest degree.

---

